# Salvage Transoral Laser Microsurgery for Radiorecurrent Laryngeal Cancer: Indications, Limits, and Outcomes

**DOI:** 10.1007/s40136-017-0143-7

**Published:** 2017-02-07

**Authors:** J. Meulemans, P. Delaere, S. Nuyts, P.M. Clement, R. Hermans, V. Vander Poorten

**Affiliations:** 1 Department of Otorhinolaryngology-Head and Neck Surgery, University Hospitals Leuven, Herestraat 49, 3000 Leuven, Belgium; 2 Department of Oncology, Section Head and Neck Oncology, KU Leuven, Leuven, Belgium; 3 Department of Radiotherapy, University Hospitals Leuven, Leuven, Belgium; 4 Department of Oncology, Section Experimental Radiotherapy, KU Leuven, Leuven, Belgium; 5 Department of Medical Oncology, University Hospitals Leuven, Leuven, Belgium; 6 Department of Radiology, University Hospitals Leuven, Leuven, Belgium

**Keywords:** Conservation surgery, Laryngeal cancer, Salvage surgery, Transoral laser microsurgery, Squamous cell carcinoma

## Abstract

**Purpose of Review:**

The aim of this report is to identify relevant literature reports on salvage transoral laser microsurgery (TLM); to consider its oncologic and functional outcomes, as well as reported complications; and to address indications and limitations of salvage TLM.

**Findings:**

The weighted average of local control after first salvage TLM was 57%. Repeated TLM procedures for second or third recurrences were required in up to 41% of cases, resulting in a weighted average of local control with TLM alone of 67%. The rate of definite laryngeal preservation was 73%. The ultimate local control rate, including cases that required total laryngectomy, was 90%. The overall complication rate after salvage TLM was 14%.

**Summary:**

Salvage TLM of radiorecurrent laryngeal cancer yields excellent oncologic outcomes. Serious complications are scarce, hospitalization times are short, and functional outcomes in terms of voice and swallowing are favorable when compared to open conservation laryngeal surgery. The key to success is an optimal patient selection.

## Introduction

Radiotherapy (RT) is widely used in the primary management of early laryngeal squamous cell carcinoma (SCC) with good functional and oncologic results. The reported rates of local control with RT alone for T1 glottic SCC range from 84 to 95% [1]. For T2 glottic tumors, local control rates between 50 and 85% have been reported [1]. Concerning early supraglottic SCC, 5-year local control rates up to 100% for T1 lesions and up to 86% for T2 tumors were achieved after primary irradiation [2, 3]. When these laryngeal cancers recur after primary radiation treatment, surgical salvage in the form of total laryngectomy (TL) has traditionally been the gold standard with good oncologic outcomes [4, 5]. However, in selected cases, salvage conservation laryngeal surgery may be considered as a more functional alternative to TL. This encompasses both open surgical techniques such as horizontal supraglottic laryngectomy, vertical partial laryngectomy, supracricoid partial laryngectomy, and extended hemilaryngectomy with tracheal autotransplantation on the one hand and transoral techniques such as transoral laser microsurgery (TLM) and transoral robotic surgery (TORS) on the other [6, 7]. Whereas open salvage conservation laryngeal surgery has good oncologic outcomes in experienced hands, its widespread use is hampered due to the technical complexity of the procedures, the lack of expertise in most centers, unpredictable functional outcomes regarding voice and swallowing, high rates of complications (fistula formation and aspiration pneumonia), and long hospitalization time [7, 8••]. On the other hand, TLM has a proven track record in the primary management of glottic and supraglottic cancer, yielding excellent local control rates comparable to primary irradiation, but the role of TLM as a salvage treatment for radiorecurrent laryngeal cancer is less elucidated [1, 9, 10, 11••]. Its first description dates from the mid-1980s, when two different low volume series of 10 and 15 patients reported local control rates of 50 and 40%, respectively [12, 13]. The aim of this report is to identify relevant literature reports on salvage TLM and to summarize its oncologic and functional outcomes, reported complications, indications, and limitations.

## Materials and Methods

The primary search for this review was conducted in September 2016 using the MEDLINE database including the literature published from 1995 to 2016. The following search strategies were used: “Salvage TLM” or “Salvage Therapy [MESH] AND Laser Therapy [MESH] AND Laryngeal Neoplasm [MESH],” yielding 20 and 37 results, respectively. Initially, no limitations on the search were imposed and peer-reviewed journals in all languages were included. Abstracts and titles were screened for relevance, and the full articles of the selected records were reviewed. The reference lists of relevant papers were scrutinized to identify additional papers of potential interest. Inclusion criteria for articles at this stage were English language, published series of 10 or more cases, transoral laser treatment of recurrent laryngeal cancer (glottic or supraglottic subsite) after primary RT or chemo-RT. After excluding papers which did not meet the listed criteria and after removing duplicates, 14 articles were finally included. Oncologic and functional outcomes and complications were recorded and analyzed, and statements concerning indications and/or limitations of salvage TLM which were based on objective results or the experience of the authors were reviewed and summarized. Weighted averages of local control and laryngeal preservation rates were calculated in this manner: for each included paper, the product of the reported average local control/laryngeal preservation rates with the number of included patients was determined. The sum of these products was calculated and subsequently divided by the total number of patients included in all papers.

## Results

Fourteen papers form the basis for this review: 13 are retrospective descriptive series and 1 is a two-center prospective case series [14], with a population size ranging between 10 and 53 subjects. The series of Puxeddu et al. [15] is a two-center retrospective case series, with results of one included center updated in a later publication by Piazza et al. [16], which, in turn, was updated by Del Bon et al. [17]. Most authors reported on series of rT1 to rT2 glottic cancer, while some authors also included more advanced glottic cases (rT3 or even rT4) [11••, 18–21] or supraglottic recurrences [14, 18, 20–22].

### Oncologic Outcomes

#### Overall Population

Oncologic results as reported in the 14 included papers are listed in Table 1 [11••, 14–26]. The follow-up ranged from 0.3 to 215 months, with the reported mean and median (depending on the series) follow-up ranging from 25.2 to 87.9 months. Local control after a first salvage TLM procedure for recurrent laryngeal cancer varied between 38 and 81%, with a weighted average among studies of 57%. One study reported on a 2-year estimated local control rate of 88% [14]. Repeated TLM procedures for a second or third recurrence were required in up to 41% of cases, resulting in local control rates with TLM alone varying between 50 and 87%, with a weighted average of 67%. The rates of definite laryngeal preservation, as obtained after TLM alone or a combination of TLM and salvage open partial laryngectomy, ranged from 50 to 94% with a weighted average of 73%. The ultimate local control rate, including cases that required salvage TL due to failure of salvage TLM, was 80 to 100%, with a weighted average of 90%. Concerning disease-specific survival (DSS) and overall survival (OS), variability in reporting is noticed, with some papers only giving the number of disease- and non-disease-related deaths during follow-up allowing for reconstruction of DSS and OS rates at the end of follow up, while other authors communicate the Kaplan-Meier estimates of 3- and/or 5-year OS and DSS. Regarding OS, 3-year estimates of 67.5 to 93.7% and 5-year estimates of 53 to 91% are reported. For DSS, 3-year and 5-year estimates both ranging between 68.6 and 100% were encountered in the included literature.

**Table 1:** Oncologic outcomes of salvage TLM for radiorecurrent laryngeal cancer

Author, year	Population size (*n*)	Tumor subsite (*n*)	rT status (*n*)	Follow-up, months, mean/median (range)	Local control at first TLM (%)	>1 laser procedure (*n*)	Local control at repeat TLM (%)	Laryngeal preservation rate (%)	Ultimate local control after salvage TL (%)	DSS	OS
Outzen et al., 1995 [18]	15	Glottis = 12 Supraglottis = 2 Subglottis = 1	rT1 = 11 rT2 = 2 rT3 = 1 rT4 = 1	35 (2–60)	73	0	–	87	80	80%	53%
Quer et al., 2000 [22]	24	Glottis = 21 Supraglottis = 3	rT1 = 18 rT2 = 6	30–126	75	0	–	75	100	100% (5 years^a^)	76% (5 years^a^)
De Gier et al., 2001 [23]	40	Glottis = 40	NA	77 (17–215)	42	3	50	50	92	92.5%	NA
Pukander et al., 2001 [26]	10	NA	NA	NA	60	NA	NA	60	100	100%	100%
Steiner et al., 2004 [11••]	34	Glottis = 34	rT1 = 11 rT2 = 10 rT3 = 10 rT4 = 3	38.6 (0.3–132.2)	38	14	71	79	85	86% (3 and 5 years^a^)	74% (3 years^a^) 53% (5 years^a^)
Puxeddu et al., 2004 [15]	16	Glottis = 16	rT1 = 12 rT2 = 4	45 (9–79)	81	1	87	94	87.5	87.5%	75%
Ansarin et al., 2007 [25]	37	Glottis = 37	rTis = 4 rT1 = 21 rT2 = 12	44 (18–88)	65	0	–	70	89	–	86% (5 years^a^)
Piazza et al., 2007 [16]	22	Glottis = 22	rT1 or rT2	NA	68	3	82	82 75 (5 years^a^)	91	95% (5 years^a^)	68% (end of FU)
Grant et al., 2008 [14]	27	Glottis/subglottis = 20 Supraglottis = 7	NA	45.6 (13.2–128.4)	88 (2 years^a^)	NA	NA	NA	NA	NA	59% (2 years^a^)
Roedel et al., 2010 [19]	53	Glottis = 53	rT1 = 45 rT2 = 5 rT3 = 1 rT4 = 2	87.9	41.5	20	58	72	77	68.6% (3 and 5 years^a^)	67.5% (3 years^a^) 53.3% (5 years^a^)
Han et al., 2012 [24]	18	Glottis = 18	rT1 = 14 rT2 = 4	49.5 (12–87)	56	1	61	67 65.1 (3 years^a^) 54.3 (5 years^a^)	89	100% (3 years^a^) 90% (5 years^a^)	93.7% (3 years^a^) 84.3% (5 years^a^)
Del Bon et al., 2012 [17]	35	Glottis = 35	rT1 = 23 rT2 = 12	NA	63	6	77 84 (5 years^a^)	80 87 (5 years^a^)	94	94% (5 years^a^)	91% (5 years^a^)
Reynolds et al., 2013 [20]	10	Glottis = 5 Supraglottis = 5	rT1 = 1 rT2 = 5 rT3 = 4	25.2 (3–56)	70	0	–	NA	NA	80%	60%
Fink et al., 2016 [21]	42	Glottis = 28 Supraglottis = 14	rT1 = 16 rT2 = 19 rT3 = 7	NA	NA	7	NA	76	100		81%

*DSS* disease-specific survival, *NA* not applicable, *OS* overall survival, *TL* total laryngectomy, *TLM* transoral laser microsurgery

^a^The Kaplan-Meier survival estimate

#### Anterior Commissure Involvement

Most studies give details about the rate of patients with anterior commissure (AC) involvement and the oncologic outcomes of this subgroup. Most authors noticed higher rates of the development of a second recurrence following salvage TLM in patients with AC involvement when compared to patients with the AC free of tumor: second recurrence rates of 50 versus 11% [18], 71 versus 48% [23], 67 versus 8% [15], and 55 versus 29% [24], respectively, were reported. On univariate analysis, Han et al. identified AC involvement as a negative prognostic factor for 3-and 5-year local control rates (51.9 versus 85.7% and 0 versus 64.2%, *p* = 0.09) [24]. This trend towards lower local control was not confirmed by data from Steiner et al. [11••] and Del Bon et al. [17]. However, Steiner et al. did observe a higher rate of salvage laryngectomies in patients with AC involvement (29 versus 8%) [11••], which was also observed by De Gier et al. (59 versus 44%) [23] and Roedel et al. (39 versus 16%) [19].

#### Early-Stage Versus Advanced-Stage Recurrences

Most authors did observe a lower local control rate in groups with advanced-stage recurrence (rT3–rT4) when compared to early-stage recurrent carcinomas (rT1–rT2). Steiner et al. demonstrated a slightly lower number of local recurrences and salvage laryngectomies in patients with early-stage recurrent carcinomas versus advanced-stage recurrences (57 versus 62%; 19 versus 23%) [11••]. Ansarin et al. observed a significantly worse estimated 5-year recurrence-free survival (RFS) rate among patients with locally advanced recurrent cancer treated with salvage TLM (rT3): he observed a 5-year RFS of 0% for rT3 patients (*n* = 4) versus 61 and 65% for rT2 and rT1 patients, respectively [25]. Interestingly, Han et al. demonstrated, within a group of early-stage recurrent glottic cancer, a significant higher 3-year and 5-year local control rates in patients with a rT1 tumor versus patients with a rT2 tumor (87.5 versus 44.4% and 87.5 versus 16.6%, *p* = 0.02). The same was true for 3-year and 5-year laryngeal preservation rates (87.5 versus 44.4% and 87.5 versus 33.3%, *p* = 0.03) [24]. Moreover, Del Bon et al. discovered on univariate analysis that the rpT category had a statistically significant impact on local control with laser alone (100% for rpT1, 67% for rpT2, and 50% for rpT3), both in the entire cohort and in the group with true persistent and recurrent disease, excluding second primary glottic carcinomas in a previously irradiated area [17]. In contrast to the compromised local control in advanced-stage cases, DSS and/or OS did not prove to be significantly different between early-stage and advanced-stage recurrences, but this may turn out to be different in larger cohorts with longer follow-up [11••, 17, 19].

### Functional Outcomes and Complications

The functional outcomes and complications after salvage TLM are outlined in Table 2. The mean hospitalization time varied between 2.2 and 9 days. Tracheotomy was not required as a routine procedure (in contrast to open partial laryngeal surgery) with most studies reporting a 0% tracheotomy rate. However, in four series [11••, 20, 21, 24], postoperative tracheotomy proved necessary in 2 to 22% of cases, with decannulation times ranging from 4 days to more than 4 months. The nasogastric tube feeding rate ranged from 0 to 23.5% with all nasogastric tubes being removed after 8 days. Three studies reported cases in which long-term gastrostomy tube (G-tube) feeding was required [19–21]. However, two of these studies, in which both glottic and supraglottic recurrences were included, reported—as expected—higher G-tube dependency in the supraglottic recurrences when compared to the glottic cancer recurrences (60 versus 20% [20] and 79 versus 21% [21]). When all patients of the included studies were taken into account (*n* = 350), postoperative tracheotomy need and G-tube requirement were only 2.3% (*n* = 8) and 6.6% (*n* = 23), respectively. Concerning vocal outcomes after salvage TLM, only limited data are available, with most studies being retrospective and stating that postoperative voice is considered to be *functional* or *acceptable* in the majority of cases. Puxeddu et al. communicated postoperative perceptual voice evaluation (grade, roughness, breathiness, asthenia, strain (GRBAS)) outcomes, showing mild, moderate, and severe dysphonia in 12.5, 25, and 37.5% of patients, respectively. Laryngostroboscopy showed an incomplete glottal closure in 56% of patients [15]. Fink et al. observed a significant increase in mean voice handicap index (VHI) (from 31.8 preoperatively to 53.5 postoperatively, *p* = 0.026) and decrease in perceptual score (from 57.0 to 45.3, *p* = 0.017) in patients who underwent salvage TLM for glottic recurrences [21]. Of interest is the functional analysis by Del Bon et al., who retrospectively compared 10 salvage TLM patients with 10 patients treated by primary TLM, after matching for age, gender, and pT category. At least 2 years after surgery, both groups were evaluated with a comprehensive voice assessment, including VHI, perceptual GRBAS scale, and objective analysis with the multidimensional voice program (MDVP). Swallowing was evaluated by completion of the M.D. Anderson Dysphagia Inventory (MDADI), fiberoptic endoscopic examination of swallowing (FEES), and videofluoroscopy (VFS). No statistically significant differences in functional outcomes were observed between the salvage and primary TLM groups [17]. However, this finding seems contradictory to the authors’ clinical feeling that functional outcome, especially concerning voice, after salvage TLM, is consistently poorer when compared with primary cases. Future comparative analyses with larger patient cohorts will be necessary to address this issue. The overall complication rate after salvage TLM amounted to 14% (*n* = 49) when all patients of the included studies were taken into account (*n* = 350). Major complications were temporary laryngeal edema (*n* = 6, 1.7%), postoperative endolaryngeal hemorrhage (*n* = 3, 0.9%), development of late laryngeal stenosis (*n* = 10, 3.5%) with subsequent late tracheotomy need (*n* = 5) or salvage TL (*n* = 1), postoperative laryngeal chondritis and/or chondronecrosis (*n* = 10, 3.5%) with subsequent tracheotomy need (*n* = 1) or salvage TL (*n* = 3), and aspiration pneumonia (*n* = 2, 0.6%). Minor complications were prolonged (>2 months) postoperative pain (*n* = 5, 1.4%), temporary numbness of the tongue (*n* = 2, 0.6%), and temporary hypoglossal nerve palsy (*n* = 1, 0.3%). Laryngeal synechiae and laryngeal granulation formation developed in seven (2%) and three (0.9%) cases, respectively, and could be managed by additional laser procedures.

**Table 2:** Functional outcomes and complications of salvage TLM for radiorecurrent laryngeal cancer

Author, year	Population size (*n*)	Mean hospitalization time (days)	Post-op tracheostomy rate and mean duration (days)	Feeding tube rate and duration	Complications	
Outzen et al., 1995 [18]	15	NA	0%	13% (all supraglottic), duration NA	Prolonged pain (*n* = 5)	
Quer et al., 2000 [22]	24	1–5	0%	12.5% (2–4 days)	Laryngeal stenosis (*n* = 2), treated with tracheotomy in 1 case 1 month after TLM	
De Gier et al., 2001 [23]	44	NA	NA	NA	Chondritis (*n* = 2) with 1 tracheostomy	
Steiner et al., 2004 [11••]	34	9	3% (52)	23.5% (2–6 days)	Aspiration pneumonia (*n* = 1), chondronecrosis and TL (*n* = 1), AC synechiae (*n* = 3), laryngeal stenosis and tracheotomy (*n* = 1)	
Puxeddu et al., 2004 [15]	16	2.2	0%	0%	None	
Ansarin et al., 2007 [25]	37	3	0%	0%	Laryngeal stenosis (*n* = 4) with 1 tracheostomy	
Piazza et al., 2007 [16]	22	4 (3–9)	NA	NA	Late chondritis (*n* = 1), late chondronecrosis (*n* = 2)	
Roedel et al., 2010 [19]	53	NA	0%	NG = 13% (8 days) G = 4% (permanent)	Postoperative endolaryngeal hemorrhage (*n* = 2) Temporary laryngeal edema (*n* = 5) Synechia (*n* = 4) Aspiration pneumonia (*n* = 1) Chondronecrosis treated with TL (*n* = 1) Laryngeal stenosis (*n* = 3) treated with tracheotomy (*n* = 2) and TL (*n* = 1)	
Han et al., 2012 [24]	18	5.5 (4–42)	22% (4–32)	0% (but NPO in 28% for 2–11 days)	Aspiration pneumonia (*n* = 1) Laryngeal tissue granulation (*n* = 3) Temporary numbness of tongue (*n* = 2) Temporary hypoglossal palsy (*n* = 1)	
Del Bon et al., 2012 [17]	35	4 (3–9)	0%	2.9% (7 days)	Major postoperative bleeding and revision (*n* = 1) Late chondritis (*n* = 1), late chondronecrosis (*n* = 2)	
Reynolds et al., 2013 [20]	10	NA	20% (1 > 4 months)	G = 40% (all >4 months)	NA	
Fink et al., 2016 [21]	42	NA	2% (duration NA)	G = 36% (9.3 months) + 5% (until death)	Postoperative laryngeal edema and tracheotomy (*n* = 1)	

*AC* anterior commissure, *G* gastrostomy tube, *NA* not applicable, *NG* nasogastric tube, *NPO* nothing per oral diet, *TL* total laryngectomy, *TLM* transoral laser microsurgery

## Discussion

### Oncologic Outcomes

When compared to open partial laryngeal surgery techniques, salvage TLM as a treatment modality for radiorecurrent laryngeal cancer combines good oncologic outcomes with the advantages of reduced complications, better functional outcomes in terms of voice and swallowing (lesser rates of tracheostomy and tube feeding), shortened hospitalization time, and superior cost-effectiveness [7, 16]. However, a trend towards inferior local control rates, even after repeat TLM, when compared to open partial laryngectomy techniques, was demonstrated in a recent systematic review and meta-analysis by Ramakrishnan et al. [8••]. When putting all the data together, failure of salvage laser treatment alone does not seem to negatively influence ultimate local control (after salvage TL) and survival (Table 1): the weighted average of local control rate with TLM alone is 67%, but the ultimate local control rate, which includes cases that required salvage TL due to failure of salvage TLM amounted to a weighted average of 90%. This high rate of ultimate local control is related to the fact that subsequent recurrences after TLM are generally contained within the larynx, which can possibly be explained by preservation of the cartilage during endolaryngeal surgery without opening the neck [25]. On the contrary, Roedel et al. did observe a significant decrease in 3- and 5-year overall (56.6 versus 81.8% and 40.2 versus 70.5%, *p* = 0.03) and DSS (48.9 versus 100% each, *p* = 0.0001) in the group of patients who developed a second recurrence after the first salvage TLM procedure [19]. The authors conclude that TL as an ultimate treatment modality for local failure after salvage TLM does not preclude a tumor-related death. Nevertheless, the same authors suggest early TL for ultimate salvage in case of failure of TLM for the first recurrence. Del Bon et al. also observed that recurrence after salvage TLM negatively influenced both organ preservation and local control with laser alone and, when excluding second primary cases, recurrence after salvage TLM also compromised OS and DSS. This knowledge prompts us to strongly consider TL or open partial laryngectomy when a new recurrence occurs after a first TLM salvage attempt, rather than going for a second salvage TLM procedure [17].

When compared to local control rates after primary TLM for laryngeal cancer, which amounts to 94% [27], local control rates after salvage TLM are inferior. This fact is likely due to different patterns of tumor growth in the radiorecurrent setting, with the presence of submucosal spread and multifocal development of recurrences, that can easily be understaged by endoscopy and imaging [19, 28]. For sure, clinical and radiological evaluation in an often still edematous larynx is more difficult. Several authors report on frequent understaging of the radiorecurrent cancer before the TLM procedure. Ansarin et al. suspected an rT2 recurrent disease in 32% of cases but eventually identified rT2 and rT3 recurrences in 49% of patients [25]. Roedel et al. reported that in 60% of patients, rT stage had been underestimated by preoperative clinical examination when compared to its true extension as seen during microsurgical exploration and resection: in 22 out of 53 patients (42%), advanced-stage recurrence (rT3 and rT4a) was evident on surgery, although this had only been assumed preoperatively in 3 patients (6%). Han et al. reported understaging in 28% of patients, and sadly but interestingly, four out of five preoperatively understaged cases recurred after the first TLM. Because post-irradiation edema and chondritis pose additional difficulties for the exact preoperative identification of tumor borders, frozen section analysis of resection margins is proposed by some authors [11••, 22]. The clinical importance of obtaining free margins was illustrated by Ansarin et al., who observed a negative influence of the presence of positive or close margins on recurrence-free survival (univariate analysis) [25]. Del Bon et al. identified the status of the surgical margins as a predictor for organ preservation and local control with laser alone, with incomplete resection with multiple superficial and/or deep positive margins negatively influencing local control and organ preservation rates, even after second look procedures [17]. Not surprisingly, extreme effort should always be made to obtain a complete resection with clear margins at the first attempt. Having this knowledge in mind, a more aggressive resection is necessary in the salvage setting and ultra-narrow margin resections (e.g., type I and II cordectomies) should be avoided in favor of wider cordectomies (type III and VI cordectomies—with low threshold to perform type V and VI cordectomies in our own experience) [17, 21]. Moreover, surgeon’s experience is considered of an extreme importance when performing salvage TLM [11••, 17, 20].

### Anterior Commissure Involvement

Obvious involvement of the anterior commissure (AC) in radiorecurrent carcinoma negatively influences recurrence rates after salvage TLM. This can be explained by laryngeal anatomy: only 2–3 mm separates the AC mucosa from the thyroid cartilage and there is no perichondrium at the level of the insertion of the AC tendons into the thyroid cartilage. As a consequence, tumors of the AC may invade the thyroid cartilage without any invasion of the vocalis muscle, and the vocal fold mobility may be preserved [18]. This may lead to understaging of tumors involving the AC, which, in combination with increased difficulty in evaluating the submucosal extension of a radiorecurrent tumor and the well-known issue of more difficult exposure, may play a crucial role in the reduced effectiveness of resection [15]. Irrespective of these anatomical considerations, the debate whether AC involvement is a contraindication for salvage TLM continues. De Gier et al. were the first to consider the extension of the recurrent tumor in the AC as a strict contraindication for salvage TLM. As the local control rate and laryngeal preservation rate of salvage TLM dropped when the AC was involved, the authors suggest choosing for an open salvage conservation surgical approach for these cases [23]. In contrast to this, Steiner did not consider the extension of the recurrence into the AC as a contraindication for salvage TLM. Actually, most patients in his series had AC involvement and no difference was observed in the second recurrence rate between patients with and without AC involvement (57 versus 54%). However, in the group of patients with compromised exposure of AC, more salvage total laryngectomies were performed (29 versus 8%), hence a lower laryngeal preservation rate in this group. He concluded that for the experienced TLM surgeon, AC involvement should not be considered an absolute contraindication to salvage TLM on the condition that tumor exposure is adequate [11••]. In line with this statement, Roedel et al. observed a comparable ultimate locoregional control and survival, be it at the expense of a higher rate of salvage laryngectomies, in the group of patients with AC involvement (39 versus 16%) [19]. In the same line, Ansarin et al. observed that preoperative suspicion of involvement of the AC had no influence on recurrence-free survival (univariate analysis; *p* = 0.45). The latter report needs to be interpreted with caution, given the fact that preoperative suspicion of AC involvement was used to define this subset of patients, rather than the actual AC involvement as observed during TLM [25]. A pragmatic approach to this issue was expressed by Piazza et al., who advised to use extreme caution during salvage TLM of lesions with involvement of the AC, in view of deep nests of tumor, invading visceral compartments or focally involving the laryngeal framework, that could compromise oncologic outcome. As a consequence, they suggested performing supracricoid partial laryngectomy (SCPL) in cases of macroscopic involvement of the AC, aiming at wider margins of resection [16]. The same research group later supported this statement by identifying anterior transcommissural extension (above and below the vocal cords) as a significant negative predictor on overall survival. However, superficial involvement of the AC limited to the glottic plane did not negatively affect oncologic outcomes or organ preservation rate [17]. In line with this, Han et al. observed that all rT1 tumors with AC involvement were cured with the first TLM, but six out of seven patients in the rT2 group with AC involvement recurred. They propose that TLM is appropriate for the treatment of rT1a and rT1b lesions involving the AC, but promote an external approach for rT2 lesions with AC involvement [24].

### Functional Outcomes and Complications

The most appealing feature of salvage TLM is that on the one hand, opening of the thyroid cartilage is avoided, thus minimizing the risk of chondronecrosis and tracheotomy, and on the other, TLM allows a microscopic tumor-adapted resection, leaving untouched as much normal larynx as possible [11••, 16, 22]. This results in few complications and superior functional outcomes in terms of voice and swallowing when compared to open partial laryngeal surgery [7]. In reports dealing with salvage TLM for radiorecurrent laryngeal carcinoma, few long-term swallowing problems were reported and voice quality was satisfactory. However, objective measures evaluating swallowing function and voice are lacking in most published papers. When vocal outcome was measured, a decrease after salvage TLM for glottic recurrences was evident [15, 21], but no significant differences were observed between a salvage and a primary TLM group [17]. As opposed to glottic recurrences, which exhibited excellent postoperative swallowing function, supraglottic location of recurrences is related to postoperative swallowing dysfunction and G-tube dependency [20, 21]. This finding is supported by Hutcheson et al., who compared in a retrospective case-control study functional outcomes of TLM in patients with previously untreated supraglottic carcinoma with post-irradiation salvage cases. Length of feeding tube dependency and rates of chronic aspiration were significantly higher in the salvage TLM group [29].

### Indications and Limits

A high variability between included papers is observed regarding indications/contraindications and selection criteria for salvage TLM of postradiation recurrent cancer. However, some generally accepted prerequisites for salvage TLM have been identified. To begin with, preoperative imaging (MR or CT) is considered obligatory by all authors, in order to exclude infiltration of the laryngeal cartilaginous framework and to quantify involvement of the paraglottic space. Next, adequate laryngeal exposure is considered as an absolute necessity for an oncologically sound transoral resection. In order to estimate the laryngeal exposure preoperatively, Piazza et al. developed a scoring system, the “Laryngoscore,” which proves to be a good predictor of difficult laryngeal exposure and assists in selecting the ideal candidates for TLM, but which awaits external validation [30•]. However, developing their scoring system, the authors identified previous RT, as well as open-neck surgical approaches, as negative prognosticators for favorable laryngeal exposure [30•]. Infiltration of the thyroid and cricoid cartilages as detectable by CT or MR and extensive infiltration of the soft tissue of the neck are generally accepted strict contraindications. Concerning rT status, advanced recurrences (rT3 and rT4) can be considered a relative contraindication, given the lower local control rates. As discussed in detail above, AC involvement is not a strict contraindication for TLM, but the surgeon needs to be aware of the technical difficulty of treating these lesions with often disappointing results, especially when gross or transcommissural involvement is present. A limited degree of subglottic extension (5 mm) of the tumor is tolerated by most authors [15, 22, 25] as well as limited supraglottic spread up to 5 mm above the free edge of the glottis [25] or not further than the lateral extension of Morgagni’s sinus [7]. The presence of normal vocal cord mobility is also considered a selection criterion by most authors [16, 22, 24], and the arytenoid (except from the vocal process) needs to be free of tumor [15, 22, 25]. However, Steiner et al. and Roedel et al. only consider fixation of both vocal cords or arytenoids a contraindication for salvage TLM [11••, 19]. Some authors consider any involvement of the paraglottic space (PGS) as a strict contraindication [16, 25], while others tolerate minimal involvement of the anterior portion of the PGS [15, 17]. Moreover, the recurrence should correlate with site and extent of the primary tumor before RT [16, 22]. Finally, the willingness of the patient to undergo a strict postoperative follow-up with regular laryngoscopic examinations and imaging, allowing for early detection and treatment of further localized recurrences, is generally considered an essential key to success [11••, 16, 17, 25]. New advances in technology such as narrow band imaging (NBI) could be beneficial in the follow-up of patients who underwent salvage TLM [31].

## Conclusion

If patients are well selected, salvage TLM of radiorecurrent laryngeal cancer yields good rates of local control with laser alone and excellent ultimate local control rates, OS and DSS, when eventual further recurrences are timely and adequately treated. Serious complications are scarce, hospitalization times are short, and functional outcomes in terms of voice and swallowing are favorable when compared to open conservation laryngeal surgery. However, the key to success is an optimal patient selection, as outcomes are influenced by a variety of patient-related and tumor-related factors.
